# Alterations in motor modules and their contribution to limitations in force control in the upper extremity after stroke

**DOI:** 10.3389/fnhum.2022.937391

**Published:** 2022-07-28

**Authors:** Gang Seo, Sang Wook Lee, Randall F. Beer, Amani Alamri, Yi-Ning Wu, Preeti Raghavan, William Z. Rymer, Jinsook Roh

**Affiliations:** ^1^Department of Biomedical Engineering, University of Houston, Houston, TX, United States; ^2^Department of Biomedical Engineering, Catholic University of America, Washington, DC, United States; ^3^Center for Applied Biomechanics and Rehabilitation Research, MedStar National Rehabilitation Hospital, Washington, DC, United States; ^4^Department of Mechanical Engineering, Korea Advanced Institute of Science and Technology, Daejeon, South Korea; ^5^Department of Physical Medicine and Rehabilitation, Northwestern University, Chicago, IL, United States; ^6^Department of Biology, Temple University, Philadelphia, PA, United States; ^7^Department of Physical Therapy and Kinesiology, University of Massachusetts Lowell, Lowell, MA, United States; ^8^Department of Physical Medicine and Rehabilitation, Johns Hopkins University, Baltimore, MD, United States; ^9^Shirley Ryan AbilityLab, Chicago, IL, United States

**Keywords:** intermuscular coordination, stroke, motor module, muscle synergy, upper extremity, feasible force direction, isometric force generation

## Abstract

The generation of isometric force at the hand can be mediated by activating a few motor modules. Stroke induces alterations in motor modules underlying steady-state isometric force generation in the human upper extremity (UE). However, how the altered motor modules impact task performance (force production) remains unclear as stroke survivors develop and converge to the three-dimensional (3D) target force. Thus, we tested whether stroke-specific motor modules would be activated from the onset of force generation and also examined how alterations in motor modules would induce changes in force representation. During 3D isometric force development, electromyographic (EMG) signals were recorded from eight major elbow and shoulder muscles in the paretic arm of 10 chronic hemispheric stroke survivors and both arms of six age-matched control participants. A non-negative matrix factorization algorithm identified motor modules in four different time windows: three “exploratory” force ramping phases (Ramps 1–3; 0–33%, 33–67%, and 67–100% of target force magnitude, respectively) and the stable force match phase (Hold). Motor module similarity and between-force coupling were examined by calculating the scalar product and Pearson correlation across the phases. To investigate the association between the end-point force representation and the activation of the motor modules, principal component analysis (PCA) and multivariate multiple linear regression analyses were applied. In addition, the force components regressed on the activation profiles of motor modules were utilized to model the feasible force direction. Both stroke and control groups developed exploratory isometric forces with a non-linear relationship between EMG and force. During the force matching, only the stroke group showed abnormal between-force coupling in medial-lateral and backward-forward and medial-lateral and downward-upward directions. In each group, the same motor modules, including the abnormal deltoid module in stroke survivors, were expressed from the beginning of force development instead of emerging during the force exploration. The PCA and the multivariate multiple linear regression analyses showed that alterations in motor modules were associated with abnormal between-force coupling and limited feasible force direction after stroke. Overall, these results suggest that alterations in intermuscular coordination contribute to the abnormal end-point force control under isometric conditions in the UE after stroke.

## Introduction

Stroke, one of the leading causes of disability worldwide, leads to motor impairments in the human upper extremity (UE) that often induce lasting dysfunction (O’Neill et al., 2008; Langhorne et al., 2009; Feigin et al., 2014). Roughly two-thirds of stroke survivors have severe deficits in UE movements, which impact their performance in activities of daily living (ADLs) and eventually, affect their quality of life (Sveen et al., 1999; Gresham et al., 2004). The common post-stroke neuromuscular deficits in UE include abnormal muscle tone (Corcos et al., 1986; Powers et al., 1989), weakness (Colebatch and Gandevia, 1989; Adams et al., 1990), and descriptive (symptomatic) muscle synergy (Dewald et al., 1995; Levin, 1996; Beer et al., 2000; Ellis et al., 2005).

Previous studies have quantified (descriptive) post-stroke muscle synergies in UE as torque or force coupling (Dewald et al., 1995; Lum et al., 1999; Reinkensmeyer et al., 2000; Dewald and Beer, 2001; Beer et al., 2004). In terms of interjoint torque coupling, stroke impairs the ability to isolate torque generation at UE joints, inducing abnormal multi-joint torque patterns. For instance, a significant increase in shoulder adduction torques in restrained more-affected UE of stroke survivors was measured during maximum voluntary torque generation in elbow extension/shoulder flexion and internal rotation (Dewald and Beer, 2001). Moreover, an increase in elbow flexion torque was observed during the generation of shoulder abduction or external rotation torques (Dewald and Beer, 2001), regardless of the limb position (Ellis et al., 2007).

In addition to these abnormal interjoint coupling patterns, abnormal force coupling in the affected UE after stroke was also characterized. When reaching forward in a horizontal plane with more-impaired UE, stroke survivors tended to generate medially directed off-axis forces (Reinkensmeyer et al., 2000), which implied abnormal coupling of forces in the forward-medial direction, and the prevalence of this abnormal force coupling was higher in the severely impaired stroke group (Lum et al., 1999). However, how the abnormal force coupling is associated with alteration in the neuromotor control after stroke remains unclear.

The change in the neuromotor control of multi-joint coordination post-stroke has been investigated in terms of intermuscular coordination, or motor modules (Taborri et al., 2018; Hong et al., 2021). To characterize intermuscular coordination post-stroke, recent studies have adopted dimensionality reduction tools such as non-negative matrix factorization (NMF) (Cheung et al., 2005; Roh et al., 2013; Ferrante et al., 2016), principal component analysis (PCA) (Reisman, 2003), and independent component analysis (ICA) (Tresch et al., 2006; Trumbower et al., 2010). The dimensionality reduction of electromyographic (EMG) signals identifies a small number of muscle groups, or motor modules, that characterize the coordinated patterns of muscle activities which can combine to produce functional motor behaviors and provide a foundation for neuromuscular control (Ting et al., 2015).

The analysis of UE motor modules has revealed that stroke induces an alteration in the spatial connection or temporal activation of motor modules (Cheung et al., 2009, 2012; Israely et al., 2018), muscle network (Houston et al., 2021), and merging or fractionation of motor modules (Cheung et al., 2012). Specifically, the altered modules involve the abnormal coupling of shoulder and elbow muscles during dynamic reaching (Massie et al., 2011; Cheung et al., 2012) and isometric torque generation (Dewald et al., 1995). Our previous study showed that stroke alters the composition of some motor modules during an isometric task. A shoulder abductor/extensor motor module, which was also called the deltoid module, included abnormal co-activation of the three heads of the deltoid muscle (anterior, middle, and posterior deltoids) during the stable force generation phase (hold phase) of three-dimensional (3D) isometric target matching in stroke (Roh et al., 2013). Furthermore, the prevalence of the abnormal motor module activation increased with the severity of post-stroke motor impairment (Roh et al., 2015). By analyzing the UE rotational angle measured during the isometric task, Roh et al. (2015) characterized the potential contribution of altered motor modules (intermuscular coordination) to the behavioral outcome. However, the relationship between the altered muscle modules of stroke survivors and their task performance (e.g., endpoint force) has not been clearly described. Do the altered modules impair the ability of stroke survivors to develop (Mercier et al., 2004) and maintain force (Roh et al., 2013)?

In this study, we aimed to examine how stroke alters the neuromuscular control during exploratory isometric force generation (i.e., no specific guidance was provided to match the 3D target forces during the ramping force phase) and assess how the altered modules are related to the task outcomes (i.e., force vector). We hypothesized that alterations in motor modules would result in abnormal force representation, limiting the intended force control and feasible force direction, throughout all phases of the isometric force generation (i.e., during both exploratory force development and stable force maintenance phases). We recorded surface EMG and 3D force signals from 10 stroke survivors with severe impairment and six age-matched neurologically intact participants during exploratory isometric target matching tasks. A non-NMF algorithm was applied to the EMG to identify the motor modules. The characteristics of motor modules and the corresponding force representation for stroke and age-matched healthy groups were quantified and analyzed, respectively.

## Materials and methods

### Participants

The data recorded from 10 stroke survivors (five males) with severe arm motor impairment [Fugl-Meyer Assessment (FMA) score < 26/66] and six age-matched, neurologically intact participants (controls; four males) were reutilized (Roh et al., 2013, 2015) for the present study. As summarized in Table 1, the stroke group age was 61.8 ± 10.0 years (mean ± STD), with a range of 58–81 years, and the control group age was 63.2 ± 7.6 years (mean ± STD), with a range of 52–73 years. Data were recorded only from the affected arm in the stroke group, whereas we recorded data from both arms, in random order, for the control group. All participants provided informed consent in accordance with the Declaration of Helsinki, with the approval of the Northwestern University Institutional Review Board.

**TABLE 1:** Participant demographics of control (*n* = 6) and stroke (*n* = 10) groups.

	Stroke (*n* = 10)		
	Mean	SD	Range
**Age (year)**	61.8	10.0	58–81
**Time after stroke onset (month)**	174.8	94.7	68–302
**Fugl-Meyer (/66)**	17.9	3.4	12–23
**Fugl-Meyer (/22)**	10.6	1.2	9–12
**Modified ashworth score (FL/EX)**	2.1/0.7	1.1/0.7	1±4/0–2
**Sex (M/F)**	5/5		
**Side affected (L/R)**	5/5		
	**Control (*n* = 6)**		
	**Mean**	**SD**	**Range**

**Age (year)**	63.2	7.6	52–73
**Sex (M/F)**	4/2		

FL, flexion; EX, extension; M, male; F, female; L, left; R, right.

### Equipment

Both the position of the hand and 3D forces at the hand [right (+Fx), left (−Fx), forward (+Fy), backward (−Fy), upward (+Fz), and downward (−Fz)] were recorded using the Multi-Axis Cartesian-based Arm Rehabilitation Machine (MACARM), a cable robot with an end-effector wired to a cubic array of eight actuators (Mayhew et al., 2005; Beer et al., 2008). MACARM also compensated the gravity force applied to the participant’s tested arm to ensure that the targeted magnitude of the actively generated force remained uniform for all force directions. A three-DOF orientation sensor (Xsens Technologies BV, Enschede, Netherlands) strapped around the upper arm was utilized to measure upper limb motion away from the parasagittal plane. All the data (hand position, forces at hand, and rotational angle of the upper limb) were sampled at a frequency of 64 Hz and stored for further analysis. The sign of the force component in the medial-lateral direction (Fx) was reversed for the data collected from left arms to facilitate subsequent comparisons involving left and right limbs.

### Electromyography

A Bagnoli eight-channel surface EMG system (Delsys Incorporated, Natick, MA, United States) was used to record EMG signals from eight muscles of the UE: brachioradialis (BRD); biceps brachii (BI); triceps brachii, long and lateral heads (TRIlong and TRIlat); deltoid, anterior, middle, and posterior fibers (AD, MD, and PD); and pectoralis major (clavicular fibers; PECTclav). Surface EMGs were recorded using electrodes applied to the belly of each muscle in parallel to the fiber direction (Hermens et al., 1999; Delagi and Perotto, 2011). EMG signals were pre-processed (x1000 amplification, 20–450 Hz band-pass filtered) and sampled at 1920 Hz before being transmitted to the base station connected to the data acquisition system.

### Experimental protocol

**FIGURE 1:**
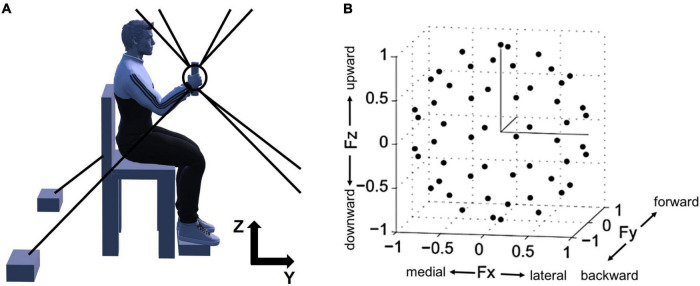
Experimental setup for isometric force measurement and normalized force targets in 3D space. **(A)** A side view of the experimental setup. For the measurement of 3D force generated at the participant’s hand, Multi-Axis Cartesian-based Arm Rehabilitation Machine (MACARM) cable-robot was used. The cable-robot consisted of a central end-effector (the circle and tilted line within the circle), a gimbaled handle (the tilted line) mounted on a six-degree-of-freedom load cell, and a spatial array of motors (two black boxes at the bottom) connected to the end-effector via cables (eight gray lines). On the top right corner, *z*- and *y*-axes of the coordinate system for the experimental setup are indicated (*x*-axis is out of the paper). **(B)** The spatial distribution of 54 normalized force targets. The end-points of normalized target force directions (54 black dots) were homogeneously distributed in 3D force space shaping an imaginary unit sphere to avoid bias of certain force directions. Fx, Fy, and Fz directions represent medial-lateral, backward-forward, and downward-upward directions, respectively.

The overall experimental setup of the study, depicted in Figure 1, was described in the previous publications (Roh et al., 2013, 2015). The participants were seated in an adjustable chair and grabbed the MACARM’s handle positioned at 60% of the participant’s arm length from the ipsilateral shoulder (Figure 1A). Additionally, to maintain the isometric condition throughout the experiment, the participant’s wrist was braced, and the trunk was strapped to the chair to restrain upper body movement. Additional strapping was used to secure the participant’s hand to the handle if he or she could not hold the handle with the affected hand.

Prior to data collection, the force sensor was zeroed to compensate for force signals associated with the weight of the limb. Subsequently, the participant’s maximum voluntary contraction (MVC) force in the lateral direction, which was the weakest among six directions across all participants, was measured. 40% of MVC was used as the target force magnitude during isometric reaching for the force matching task.

After a short practice session, participants were instructed to perform the force-target-matching task by applying a voluntary isometric force on the handle. The 54 targets were approximately uniformly distributed in 3D force space (Figure 1B) to remove any bias in target force directions and maximize the variability of EMG data. The force targets were provided to participants in random order. Each trial started with a 2-s baseline recording period followed by the period allowed to achieve a target match (7 and 9 s for control and stroke groups, respectively). A successful target match required the participant to maintain the center of a cursor in the target zone (a sphere around the target force with a radius equal to 20% of the targeted force magnitude) for 0.8 s. Three attempts per target were given to the participants, and if they failed all the attempts, the target was recorded as unmatched. During the initial complete set of trials, the participant was required to maintain the limb in a vertical plane, with adherence confirmed based on data provided by an Xsens orientation sensor (Roh et al., 2013). Trials with substantial out-of-plane limb rotation were considered failed attempts. After the initial set of trials, participants performed three attempts for each unmatched target without the constraint on limb rotation. Between trials and 10-trial blocks, there were 10-s and 1-min breaks, respectively, to minimize muscle fatigue.

### Data analysis

For the EMG data analysis, first, the electrocardiogram (ECG) noise embedded in the EMG signals, especially in PECTclav, was removed using a wave decomposition and reconstruction method (von Tscharner et al., 2011). Following ECG filtering, each EMG data set was demeaned to remove any DC component from the signal. The demeaned data were then full-wave rectified, and their pre-recorded baseline values were subtracted. Any negative components resulting from the baseline subtraction were zeroed out before the entire data were further processed with a Butterworth low-pass filter (4th order, 10 Hz cutoff frequency). For the raw data of each force component (Fx, Fy, and Fz), the mean amplitude of the baseline period was subtracted to compensate for any residual offset in each trial. At the end of data post-processing, a matrix with a dimension of eight, the number of muscles recorded per each trial, by the number of data points was prepared for motor module identification.

**FIGURE 2:**
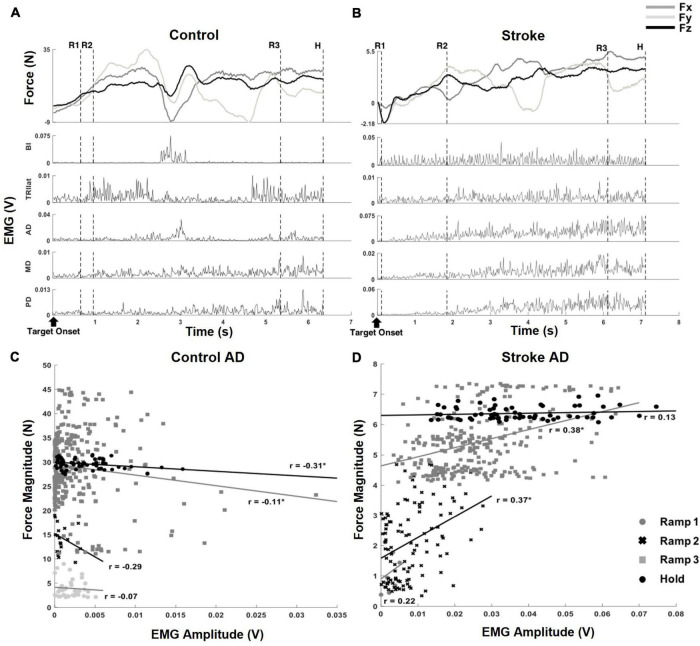
Exemplary target matching trials, aligned to the target onset, which showed how force and EMG data were parsed into segments and correlation coefficients (*r*-value) of muscle activation and output force at the hand, from a representative dataset of each group. **(A)** An exemplary target matching trial of a control participant. The segmentation of the force and EMG data (depicted by the dotted, vertical lines) was based on the relative proportion of target force magnitude achieved within each trial: (1) Ramp 1 (R1), from zero to the beginning of the first 33%, (2) Ramp 2 (R2), from the first 33% to the beginning of the 67%, (3) Ramp 3 (R3), from the beginning of the first 67% to the target match (100% amplitude of force target), and (4) Hold (H), 0.8 s holding of target force. Note that the magnitude of the end-point force components did not linearly scale up during sequential force development phases. **(B)** An exemplary target matching trial of a stroke participant. The trial with the same target direction of panel **(A)** was used for the comparison. Note that a more extended time was required for the stroke participant to match the same target, and the magnitude of force was significantly smaller than that of the control participant. In addition, the overall trend of the force development differed from the control participant. Subsequent EMG plots highlight the typical abnormal co-activation of AD, MD, and PD in stroke. **(C,D)** The correlation coefficients (*r*; *p < 0.05) of AD muscle activation and end-point force that corresponded to the trials in panels **(A,B)**.

Since there was a difference in the data acquisition rate (1920 Hz and 64 Hz for EMG and force data, respectively), the processed EMG was averaged per force data point to match the length of the force amplitude vector. Following the resampling process, both EMG and force data were segmented into four epochs to characterize the sequential force development: Ramp 1, Ramp 2, Ramp 3, and Hold (Figure 2; Roh et al., 2019).

The force and EMG segmentation was done based on the intervals between a pair of two time points in the force data. Ramp 1 segment was defined as a period between 70 ms before the onset of end-point force and the point where the force magnitude reached 33% of the target force magnitude for the first time. The time when the force magnitude became greater than two standard deviations from the average baseline amplitude was considered the onset of end-point force. The 70 ms was used to account for the electromechanical delay between the EMG onset and the onset of end-point force (Aagaard et al., 2002). Ramp 2 indicated a period between the end-point of Ramp 1 and a point where 67% of target force magnitude was first achieved. Similarly, Ramp 3 contained all data points from the end of Ramp 2 to the time when the force-driven cursor encountered the target sphere (target match) for the first time. Finally, the Hold period included the 0.8-s of data points recorded. At the same time, the cursor stayed within the target match zone (a circle with the center at the target force and the radius of 20% of the target force magnitude).

To explore the degree of the linear relationship between the activation of a muscle and the end-point force magnitude for each epoch of each trial, the Pearson correlation coefficient (*r*-value) and its statistical significance (*t*-test; alpha = 0.05) were computed from the processed EMG and force data.

### Motor module identification

We applied a non-NMF algorithm (Lee and Seung, 1999, 2000) to the normalized EMG datasets to identify the motor module vectors and their activation profile. For each epoch, the EMG datasets of all matched trials were concatenated and normalized to have a unit variance to prevent the identification of motor modules biased toward muscles with a large variance. In the NMF process, EMGs were modeled as a linear combination of a set of N motor modules and the relative activation coefficient of each module (Cheung et al., 2005, 2012; d’Avella and Bizzi, 2005; Roh et al., 2011, 2012).


(1) $${\mathrm{EMG}}_{\mathrm{subject}}\,\left(t\right)=W_{\mathrm{subject}}\cdot C_{\mathrm{subject}}\,\left(t\right)$$

W was an eight (the total number of muscles) by N matrix of motor module vectors, while C was the motor module activation profile (coefficients), a matrix whose dimension was N by the total number of EMG data points. The number of motor modules in the model, N, varied from one to eight. Motor module vectors and their activation profiles were identified from a subset of given EMG data, comprised of randomly selected 60% of the data, and used to reconstruct the remaining 40% data (Roh et al., 2012, 2013). For a given N, the identification of motor module vectors and their activation profiles was repeated 100 times. The motor modules with the highest global variance-accounted-for (gVAF) value were selected among the 100 sets for further analysis.

To identify the appropriate number of motor modules that could explain most of the total variance of any given EMG dataset, gVAF values were calculated, reflecting how the activation of all eight muscles as a group would be predicted. We also considered how each muscle activation would be predicted by a set of motor modules (mVAF) to evaluate how the nuances of the EMG data would be predicted. Total variation in the data, defined by the trace of the covariance of the EMG-data matrix, was used to calculate a multivariate VAF measure:


(2) $$\mathrm{VAF}=100\times \left(1-\frac{\mathrm{SSE}}{\mathrm{SST}}\right)$$

where SSE is the summation of the square residuals, and SST is the sum of the squared of uncentered EMG data (Zar, 2010). In addition to gVAF and mVAF values, we also accounted for the difference in gVAF when one additional module was added (diffVAF) to determine the number of motor modules per dataset. Thus, the criteria applied to estimate the number of modules for each dataset consisted of finding the minimum number of modules needed to have gVAF > 90%, mVAF > 60%, and diffVAF < 5%. The criteria ensured that the estimated number of modules would appropriately predict both the global features and nuances of EMG data.

### Motor module similarity

Using motor module vectors in the Hold period as a reference, we quantified the similarity (*r*-value) of motor modules underlying different epochs of force development (i.e., Ramps 1–3 and Hold) by calculating the scalar products of the motor module vectors of each epoch with the corresponding Hold period module. The similarity of motor module vectors was also calculated between the stroke and control groups.

To test the statistical significance of the similarity, randomly selected muscle weights of motor module vectors identified in the study were used to form 1000 sets of one by eight random synergy vectors. By calculating the similarity of all possible pairs of random synergy vectors through the scalar product, the 95th percentile of the similarity indices was identified as a similarity threshold, 0.86 (Roh et al., 2012, 2013). Any pair of motor module vectors whose similarity index (*r*-value) exceeded the similarity threshold were considered statistically similar.

### End-point force–force coupling

The coupling of force components (Fx, Fy, and Fz) for each epoch was quantified using the pair-wise Pearson correlation coefficient. Before computing the correlation, the segmented force data were concatenated across the 54 trials. Since the 54 targets were evenly spaced around the unit sphere in 3D force space, the correlation between any pair of force components, on average, would be close to zero if the participants directly matched all the targets. The computed correlation coefficient was then averaged across the participants within each group, and the difference in the mean value was statistically tested using the *t*-test (alpha = 0.05).

### Relationship between end-point force and the activation profile of motor modules

Principal component analysis method was applied to investigate the association between the force components (Fx, Fy, and Fz) and the activation profiles (C) of motor modules per individual epoch of force development. Prior to the PCA, the segmented (by epoch) force and activation profile of each motor module of each participant were normalized with their Z-scores. They formed a matrix, *M*_*PCA*_ (number of F and C components by number of data points). Using a singular value decomposition (SVD) algorithm built-in MATLAB, the principal components (PCs), consisting of the loading coefficient of the variables (F and C) and their corresponding score vectors, were extracted from *M*_*PCA*_. The loading coefficients represented the correlations of different combinations of the variables. The composition and VAF of PCs identified per epoch were compared across the participants and groups.

In addition to PCA, a multivariate multiple linear regression method was utilized to further evaluate the group’s overall force representation in terms of each motor module’s activation profiles using the regression coefficients (β).


(3) $$F_{x,y,z}\,\left(t\right)=\beta_{0}+\beta_{1}\,C_{1}\,\left(t\right)+\ldots \,\beta_{N}\,C_{N}\,\left(t\right)$$

For each epoch, the normalized force and activation profiles of motor modules were concatenated across all the participants in each group prior to the regression. The regression coefficients obtained from the regression were compared across different epochs. Note that the vector, [β_*i*_*x*,β_*i*_*y*,β_*i*_*z*],*i* = 1:*N*, represents the end-point force resulting from unit activation of motor module *i*.

### End-point force representation and feasible force direction

To model feasible force directions for each group, the force and the activation profile across all the epochs for all participants were combined for force-activation profile multivariate multiple regression. The computed regression coefficients (β) of each group were then multiplied by a set of arbitrary simulation indexes (*I*_*sim*_ = 1:100) to reconstruct the associated force magnitude and direction, which were combined to define the feasible force directions for each group.


(4) $$\mathrm{Feasible}\,\mathrm{Force}\,{\mathrm{Direction}}_{x,y,z}\,\left(I_{\mathrm{sim}}\right)=\beta_{1}\,I_{\mathrm{sim}}+\ldots \,\beta_{N}\,I_{\mathrm{sim}},I_{\mathrm{sim}}=1:100$$

## Results

### Characteristics of muscle coordination and force-electromyographic relationship during exploratory force development in control and stroke groups

Since an exploratory isometric reaching task was performed with neither restriction nor guidance on how to match the 3D force target, the participants in both control and stroke groups developed the endpoint force non-linearly throughout each trial of force target match (Figure 2). For a given target force direction, the overall force magnitude the stroke group generated (9.38 N ± 5.07 N; mean ± STD, *n* = 10) was smaller compared to that of the control group (22.38 N ± 9.98 N; mean ± STD, *n* = 12; *p* < 0.001). In addition, the average duration of target matching was also significantly different between the two groups [3.39 s ± 1.26 s and 4.64 s ± 1.62 s for control and stroke groups, respectively (mean ± STD), *p* < 0.05]. However, Ramp 3 accounted for the largest portion of the total duration in both groups (see Section “Similarities and differences in force coupling and behavioral characteristics between the control and stroke groups” for details). Though both groups shared a similar strategy for the exploratory force reaching, the corresponding activation of muscles measured from each group was coordinated differently. As shown in the representative data obtained during the same directional force target matching (Figures 2A,B), the abnormal co-activation of AD, MD, and PD gradually increased in stroke participants. In contrast, the activation of the same muscles in controls remained relatively silent.

Based on the correlation analysis at each phase, the non-linearity in the EMG-force relationship was observed in both control and stroke groups (Figures 2C,D, respectively). Across the sequential ramp phases, the correlation coefficient of the EMG signal of a muscle (e.g., AD) and the end-point force magnitude was not consistent in the both groups [For the example trial shown in Figure 2, the correlation coefficients are 0.35 (Ramp 1), 0.022 (Ramp 2), −0.041 (Ramp 3), and −0.16 (Hold) for control group and 0.49 (Ramp 1), 0.0098 (Ramp 2), 0.0109 (Ramp 3), and −0.026 (Hold) for stroke group]. As evidenced by a low *r*-value, particularly during the earlier phases, the participant tended to explore the force space rather than approaching straight to the target, which would be expected to yield fairly strong correlations between EMG and force amplitude. For the stroke group, the overall trend of change in correlation between AD muscle activation and the force was different from the control due to the co-activation of deltoid muscles. However, the non-linearity in the correlation was shown.

### Similarities and differences in force coupling and behavioral characteristics between the control and stroke groups

**FIGURE 3:**
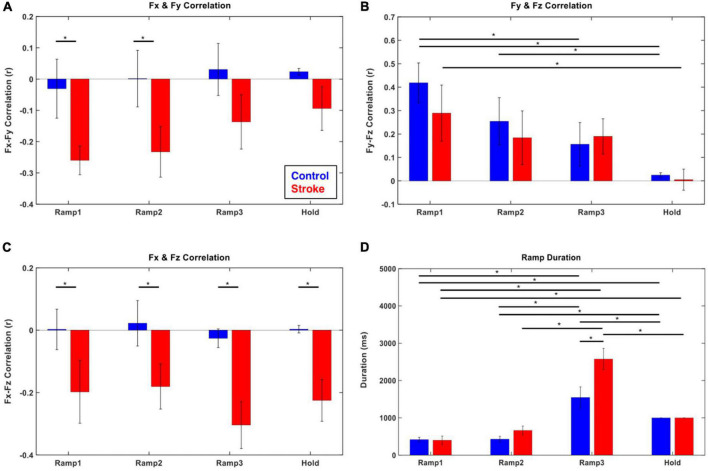
Force–Force correlation and temporal duration at each force development phase (blue: control, red: stroke). **(A–C)** The magnitude of force–force couplings was larger in stroke than that in the control. In particular, Fx and Fy (medial-lateral and backward-forward) and Fx and Fz (medial-lateral and downward-upward) directional forces were negatively correlated throughout the isometric reaching task in stroke. **(D)** The characteristics of force development strategy in terms of temporal duration of each epoch were similar between control and stroke groups. The participants spent significantly longer time in Ramp 3 compared to the other epochs. The asterisk (*) with a bar indicates the significant difference in the mean of two subgroups (epoch-epoch pair within the same group and control-stroke pair at each epoch) at the alpha level of 0.0025. Each error bar designates ± two standard errors. Bonferroni correction was applied for all the statistical analyses.

The coupling of the end-point force components (Fx-Fy, Fy-Fz, and Fx-Fz) was different between the control and stroke groups (Figures 3A–C). For the control participants, no significant correlation between the forces was observed in the transverse plane (Fx-Fy) and coronal plane (Fx-Fz). In contrast, a consistent negative correlation of force components across the epochs was found in those two planes in stroke participants (*p* < 0.05). For the force representation in the sagittal plane (Fy-Fz pair) in both groups, a positive coupling developed from the initiation of isometric reaching (Ramp 1), which vanished as the later phase was approached.

Both groups generally utilized a similar strategy to develop exploratory 3D force vectors during isometric reaching. The magnitude of the end-point force rapidly increased to about 70% of the target force magnitude (Ramps 1 and 2) first, then significantly longer time was spent fine-tuning the force direction in the following phase (Ramp 3) until the force target was reached. The duration of the Ramp 3 phase was significantly longer than that of the previous two phases (Ramps 1 and 2) for both groups (*p* < 0.05, respectively; Figure 3D). Based on the comparison between the first five and the last five of 54 trials, the difference in the duration of Ramp 3 between the control and stroke groups was smaller in the earlier trials and there was a statistically significant reduction in Ramp 3 duration in the control group (*p* < 0.05).

### Motor modules were preserved, but their activation profiles varied during exploratory force development

**FIGURE 4:**
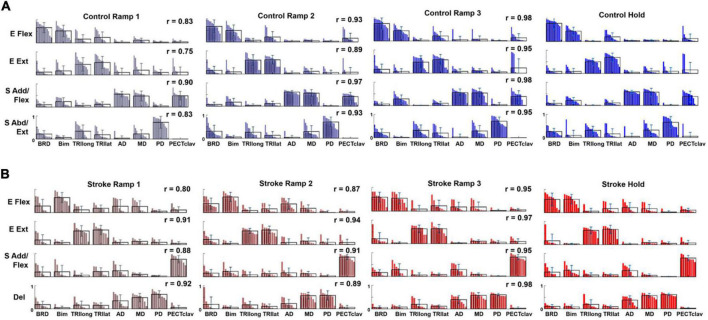
Motor modules identified and ordered across different epochs of force development in control and stroke groups **(A,B)**, respectively; (E Flex, elbow flexor; E Ext, elbow extensor; S Add/Flex, shoulder adductor/flexor; S Abd/Ext, shoulder abductor/extensor; Del, deltoid module). Each bar represents a muscle weight within a module per participant. The bars are displayed in descending order, and the mean and standard deviation are superimposed on the weight distribution of each muscle for each group. Stroke-specific motor modules were activated from the onset of force development. The number and composition of modules were conserved across different epochs of isometric reaching in control and stroke groups, respectively. The similarity between each epoch and the hold period (*r*-value) was calculated for each group.

For both control and stroke groups (Figures 4A,B, respectively), typically, four modules were identified [Control: 4.17 ± 0.39 (mean ± STD), *n* = 12; Stroke: 4.25 ± 0.46, *n* = 10] given the criteria to estimate the appropriate number of motor modules. With four modules, gVAF was 92.35 ± 1.32% and 92.93 ± 0.93% (mean ± STD) across the different phases for control and stroke groups, respectively. In terms of the composition, the four motor modules of the control group were characterized as (1) elbow flexor (E Flex), (2) elbow extensor (E Ext), (3) shoulder adductor/flexor (S Add/Flex), and (4) shoulder abductor/extensor (S Abd/Ext). E Flex module consisted of the dominant co-activation of BRD and BI, while E Ext was represented with the activation of triceps brachii heads (TRIlong and TRIlat). For the shoulder modules, the co-activation of AD, MD, and PECTclav composed the S Add/Flex, and PD was the major muscle of S Abd/Ext (Figure 4A). Compared to the control group, modules of the stroke group showed similar structures of E Flex (0.88 ± 0.09) and E Ext (0.97 ± 0.02). However, S Add/Flex consisted of dominant activation of PECTclav, while AD and MD were co-activated with PD to form the Del module (Figure 4B), which was identified as a stroke-specific module in the previous studies (Roh et al., 2013, 2015). The similarity of module composition between S Add/Flex of control and stroke group was 0.68 ± 0.13 and the similarity between S Abd/Ext and Del module was 0.80 ± 0.06, which indicated significant differences in the composition of motor modules. Based on the comparison between Hold period and each ramp, the average similarity index of all modules for Ramp 2 (control: 0.93 ± 0.032, stroke: 0.90 ± 0.030) and Ramp 3 (control: 0.97 ± 0.017, stroke: 0.96 ± 0.015) exceeded the similarity threshold in both groups. In Ramp 1, control and stroke groups showed an average similarity index of 0.83 ± 0.061 and 0.88 ± 0.054, respectively. The Ramp 1 similarity index of the control group was lower than the threshold, which implied that the low signal-to-noise ratio of EMGs recorded in Ramp 1 might affect the variability of motor module composition. However, the similarity score in Ramp 1 was not too low (0.83, on average), indicating that the composition of the four motor modules was generally consistent throughout the task, particularly in the later phases, in both groups.

**FIGURE 5:**
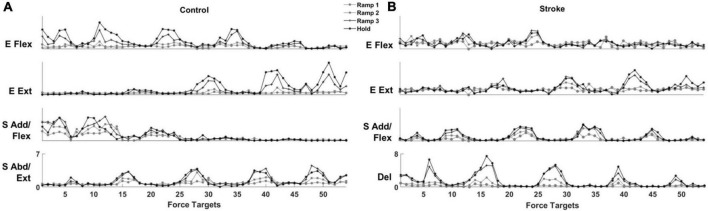
The activation profile of motor modules is modulated to meet task requirements in control and stroke groups, respectively. The averaged activation profiles of their corresponding motor modules were displayed as a function of 54 force targets at each of four force development phases (Ramp 1, 2, and 3 and Hold) in a representative control **(A)** and stroke **(B)** participant, respectively.

The activation profile of each module vector was modulated over the epochs of force development to meet task requirements. In general, the activation profile of motor modules increased as the end-point force magnitude reached the target force amplitude. During the Hold period, the average magnitude of activation profile across the participants within each group was statistically greater than during the earlier phase of the force generation, such as Ramp 1, for all four modules (control Ramp 1: 0.64 ± 0.30, stroke Ramp 1: 0.59 ± 0.38, control Hold: 1.36 ± 0.32, stroke Hold: 1.10 ± 0.31; *p* < 0.05, *t*-test). Throughout the 54 trials, the motor modules cooperated systematically to generate the end-point force in target directions. For instance, the results from a representative control participant (Figure 5A) showed that a gradual ramp-up of E Flex and S Add/Flex module activation in the fourth target matching. For the same target matching, antagonistic modules, E Ext and S Abd/Ext, remained silent throughout the trial. Since the fourth target was positioned in a medial and upward direction in force space, which required elbow flexion and shoulder adduction/flexion torques simultaneously, it was shown that the dynamic changes in the activation profile of each motor module underlay the force development trajectory. In stroke, the same tendency of modulating the activation profiles was observed. However, due to the stroke-induced alteration of intermuscular coordination, the way motor modules interacted with each other was different from the controls (Figure 5B). Based on the result of a representative stroke participant, for example, only S Add/Flex was dominantly activated in target matching to the medial-backward-downward direction (e.g., the 35th target). In contrast, E Flex was dominant in the case of the control participant (Figure 5A).

### Characteristics of the relationship between end-point force and motor module activation profiles

The PCA results showed that the first four PCs, in total, accounted for 80–90% of the variance in the data (VAF) for both control and stroke groups across the phases, respectively, when the PCs were identified from the matrix that consisted of three force components and four motor module activation profiles of each participant (see Section “Relationship between end-point force and the activation Profile of motor modules”). On average, the composition of each PC per epoch, compared to the counterpart of Hold phase, was consistent throughout the trial in both groups [*r*-value (mean ± STD); control: PC1_*CTR*_ = 0.88 ± 0.087, PC2_*CTR*_ = 0.93 ± 0.07, and PC3_*CTR*_ = 0.91 ± 0.11; stroke: PC1_*ST*_ = 0.92 ± 0.016, PC2_*ST*_ = 0.95 ± 0.026, and PC3_*ST*_ = 0.98 ± 0.12]. The VAF of PC1 in both groups increased significantly during the force generation (*t*-test; *p* < 0.05).

**FIGURE 6:**
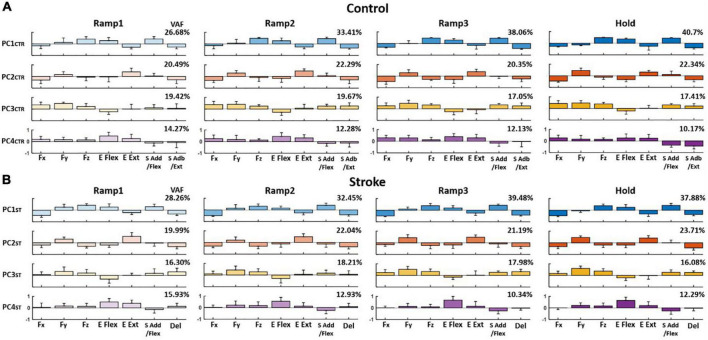
Average loading coefficients of principal components (PCs) identified from force (Fx, Fy, and Fz) and activation coefficients of each motor module (C) across four different epochs of force development in control and stroke groups **(A,B)**, respectively. For both groups, the first four PCs, in total, accounted for 80–90% of the total variance in the data. The four PCs from the control group (PC_*CTR*_) represent the correlations of a different combination of the components: PC1_*CTR*_: Fz, E Flex, and S Add/Flex; PC2_*CTR*_: Fy and E Ext, as well as Fx, E Flex, and S Abd/Ext; PC3_*CTR*_: Fx, Fy, Fz, S Add/Flex, and S Abd/Ext; and PC4_*CTR*_: Fx and Fy with E Flex and E Ext as well as S Add/Flex and S Abd/Ext. For the stroke group, each PC is represented with dominant co-activation of the force and activation profiles in the following way: Fz, E Flex, and S Add/Flex as well as Fx, E Ext, and Del (PC1_*ST*_); Fy and E Ext (PC2_*ST*_); Fy, Fz, S Add/Flex, and Del (PC3_*ST*_); and minor Fy and Fz with E Flex (PC4_*ST*_). The group average VAF of each PC was indicated at the right upper corner of each PC for each group.

When comparing the groups, the composition of PC in the stroke group was partially distinct from the control group. For example, for control, PC1_*CTR*_ included major co-activation of Fz, E Flex, and S Add/Flex, while PC2_*CTR*_ was dominated by the co-activation of Fy and E Ext as well as Fx, E Flex, and S Abd/Ext. PC3_*CTR*_ included the activation of Fx, Fy, Fz, S Add/Flex, and S Abd/Ext, and PC4_*CTR*_ was characterized by the activation of Fx and Fy with E Flex and E Ext/S Add/Flex, and S Abd/Ext (Figure 6A). For the stroke group, each PC was represented with dominant co-activation of end-point force components and activation profiles of motor modules in the following way: Fz, E Flex, and S Add/Flex, as well as Fx, E Ext, and Del (PC1_*ST*_); Fy and E Ext (PC2_*ST*_); Fy, Fz, S Add/Flex, and Del (PC3_*ST*_); and E Flex (PC4_*ST*_) (Figure 6B). The composition of PC1 and PC2 was similar between the two groups (*p* < 0.05). However, unlike the control group, PC3 in the stroke group showed a weak correlation between Fx and the shoulder modules (S Add/Flex and Del), which correlated with Fy and Fz.

Similar to the results from PCA, the overall force representation of stroke-induced modules, represented by regression coefficients (β), was distinct from the control group (Table 2). The two elbow modules of the control group antagonistically represented the force in the forward-backward direction (E Flex: −Fy, E Ext: +Fy) and in the upward-downward direction (E Flex: +Fz, E Ext: −Fz), while both represented medial force, −Fx. Moreover, the shoulder modules of the control group antagonistically represented the force in the lateral-medical direction (S Add/Flex: −Fx, S Abd/Ext: +Fx) and the upward-downward direction (S Add/Flex: +Fz, S Abd/Ext: −Fz), as well as the forward-backward direction (S Add/Flex: +Fy, S Abd/Ext: −Fy) throughout the isometric reaches. Since the muscles were synergistically coordinated as a motor module, and each module corresponded to the representation of certain force component(s), alteration of the intermuscular coordination subsequently induced changes in the end-point force representation. Thus, the end-point force representation of stroke-induced modules was altered throughout different epochs of force development. The noticeable difference included that the Del module, comparable to S Abd/Ext module in controls, did not strongly contribute to the force representation in the upward-downward direction (Fz). S Add/Flex module had a weaker contribution in the forward (+Fy) and upward (+Fz) force direction from the early stage of the force development. In addition, unlike the E Ext module of the control group, E Ext module of the stroke group corresponded to the force in the lateral direction (+Fx). Also, E Flex module did not show a strong contribution in either lateral or medial direction in stroke.

**TABLE 2:** The summary of force-activation coefficient linear regression [regression coefficients (β)] at each epoch per each of control and stroke groups.

	Control											
	**Ramp 1**			**Ramp 2**			**Ramp 3**			**Hold**		
	***Fx***	***Fy***	***Fz***	***Fx***	***Fy***	***Fz***	***Fx***	***Fy***	***Fz***	***Fx***	***Fy***	***Fz***

*E Flex*	−0.060	−0.12	0.28	−0.054	−0.29	0.38	−0.13	−0.58	0.572	−0.15	−1.2	0.57
*E Ext*	−0.18	0.11	−0.23	−0.029	0.63	−0.63	−0.052	0.48	−0.62	−0.03	0.53	−0.66
*S Add/Flex*	−0.13	0.15	0.34	−0.24	0.39	0.87	−0.38	0.62	1.1	−0.39	0.46	1.1
*S Abd/Ext*	0.28	−0.16	−0.11	0.88	−0.17	−0.28	0.96	−0.057	−0.38	0.97	−0.14	−0.40
	**Stroke**											

	**Ramp 1**			**Ramp 2**			**Ramp 3**			**Hold**		
	***Fx***	***Fy***	***Fz***	***Fx***	***Fy***	***Fz***	***Fx***	***Fy***	***Fz***	***Fx***	***Fy***	***Fz***

*E Flex*	−0.037	−0.010	0.059	−0.054	−0.086	0.16	−0.0040	−0.13	0.33	0.028	−0.24	0.34
*E Ext*	0.034	0.035	−0.084	0.057	0.17	−0.17	0.087	0.14	−0.23	0.12	0.28	−0.27
*S Add/Flex*	−0.088	0.062	0.16	−0.17	0.12	0.34	−0.27	0.22	0.43	−0.30	0.026	0.48
*Del*	0.26	−0.073	0.034	0.50	−0.087	0.094	0.53	−0.07	0.037	0.60	−0.12	0.098

In terms of the β, the force representation of each module in control participants was consistent throughout the reaching task (E Flex, −Fx and −Fy and +Fz; E Ext, −Fx and +Fy and −Fz; S Add/Flex, −Fx and +Fy and +Fz; and S Abd/Ext, +Fx and −Fy and −Fz). Across different epochs, the stroke-specific motor modules represented each force components similarly except E Flex, which changed its representation of Fx from −Fx to +Fx at the holding period (For Ramps 1–3, E Flex, −Fx and −Fy and +Fz; E Ext, +Fx and +Fy and −Fz; S Add/Flex, −Fx and +Fy and +Fz; and Del, +Fx and −Fy and +Fz).

### Alterations in feasible force direction after stroke

**FIGURE 7:**
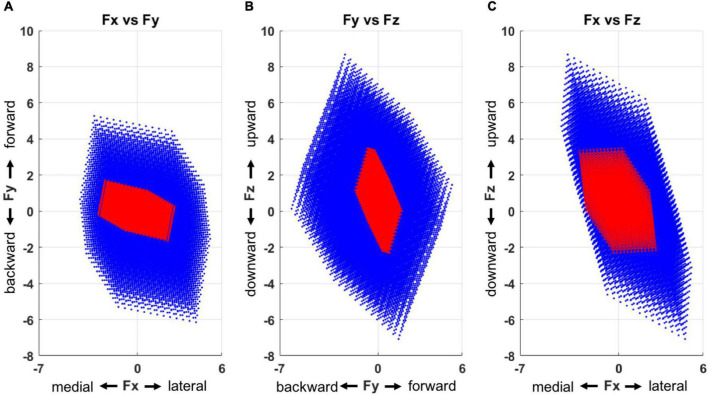
Force space representation for each group (blue: control, red: stroke), presented in three 2-dimensional force spaces (**A**, Fx vs. Fy; **B**, Fy vs. Fz; and **C**, Fx vs. Fz). Compared to the control group, the motor modules of the stroke group represented a smaller force space with a skewed directionality. The force space of the stroke group had a limited size of force sub-space in a combination of lateral, forward, and upward directions as well as medial, backward, and downward ones.

The feasible force directions, simulated based on the output of the multiple linear regression (Figure 7), depicted that stroke-induced motor modules represented a smaller and more limited sub-force space, a combination of lateral, forward, and upward directions as well as medial, backward, and downward ones from the initiation of the force generation. In total, the force targets that corresponded to 40% of unmatched trials were located out of these limited feasible force directions across the stroke participants.

## Discussion

The current study examined how alterations in motor modules after stroke relate to impaired force representation that limits the feasible force direction in 3D force space throughout exploratory isometric force target matches. Both stroke and control groups developed exploratory isometric forces with non-linear EMG-force correlation. Both groups matched the target force by using a similar behavioral strategy: increasing the force magnitude and direction accuracy at the earlier and later epochs of the force development, respectively. However, only the stroke group expressed alterations in the between-force coupling and the composition of motor modules, which appeared from the beginning of the force development instead of emerging during the force exploration. Specifically, lateral-forward and lateral-upward directional force components were negatively correlated in stroke. The PCA analysis and multivariate multiple linear regression revealed that activation of the altered shoulder modules contributed to the limited, feasible end-point force direction after stroke. Overall, these findings suggest that stroke can induce alterations in motor modules, which contributes to the degradation in intended force control and feasible force direction throughout the exploratory phases of the isometric force generation.

The finding on the conservation of altered structure of motor modules throughout the exploratory phases extends the results of our previous work (Roh et al., 2013) that reported the alterations of intermuscular coordination during the stable force generation period only. If the same muscular control under isometric conditions is applied to movement, one can reason that merging or fractionation of motor modules during reaching (Cheung et al., 2012; Park et al., 2021) may also occur from the beginning of a movement, instead of converging to the solution in the middle of a movement.

Along with the composition of motor modules, the number of modules was consistent across the epochs of force development in both groups. The observation that stroke does not induce alterations in the number of modules during exploratory force development under isometric conditions (four modules for both groups when activation of eight muscles was recorded) is consistent with the findings from other studies with a similar experimental protocol: dynamic planer reaching with gravity compensation (Tropea et al., 2013) and isometric planer reaching with gravity compensation (Pellegrino et al., 2021). A few previous studies have shown a negative correlation between the level of motor impairment and the number of motor modules compared to able-bodied controls (Clark et al., 2010; Hesam-Shariati et al., 2017). However, these studies focused on the dynamic motion of a limb without any gravity compensation, which implies that the difference in the number of motor modules between stroke and control group tends to be minute when the task involves constrained limb movement with compensation of gravity. Although the composition and the number of the motor modules remained consistent across the different phases of the isometric reaching, the activation amplitude of each module, in general, tended to increase as the participant approached the solution. This observation may imply a positive correlation between the level of motor module activation and the endpoint force amplitude, which was not observed in a single muscle EMG-endpoint force relationship.

The PCA results showed that abnormal between-force coupling was related to the activation of the altered motor modules in stroke. Based on the results from the PCA, both groups showed a similar structure of PC1 and PC2, which represented negative coupling between Fx (the direction from medial to lateral) and Fz (the direction from down to up) and between Fx and Fy (the direction from backward to forward), respectively. However, only PC3 of the control group had a positive correlation among Fx, Fy, and Fz, associated with activation of shoulder motor modules, which may compensate for the negative force coupling involved in PC1 and PC2. In stroke, the activation profiles of shoulder modules, including Del module, in PC3 were correlated with Fy and Fz without Fx, which failed to compromise the negative correlation in lateral-forward and lateral-upward force components throughout the force generation phases (Figures 3A,C). This result extends the finding from Pellegrino et al. (2021) which indicated that the activation profiles of motor modules, mainly involving proximal muscles of the stroke-affected arm, were altered when generating isometric force in lateral directions (Pellegrino et al., 2021). The emergence of the abnormal motor module with the co-activation of three heads of the deltoid after stroke suggests that stroke survivors lose the ability to control their muscle activation in isolation. The failure of isolating the PD activation, mainly in charge of shoulder abduction/extension in healthy participants, and the singled-out activation of PECTclav, a major shoulder flexor muscle, possibly induce the strong negative correlation in lateral-forward and lateral-upward force components which may limit motor function in lateral-forward-upward direction after stroke.

In addition, the results from multiple linear regression of endpoint forces and activation coefficients of motor modules also indicate that the end-point force representation of stroke-specific modules was different from that of the control group throughout different epochs. Noticeably, the contribution of the shoulder adduction/flexion module on the endpoint force in the forward (+Fy) and upward (+Fz) direction was unsubstantial compared to the corresponding motor module of the control. Furthermore, the stroke-induced deltoid module showed minimal intervention in upward-downward force (Fz) generation, mainly controlled by the control group’s the shoulder abduction/extension module.

The feasible force direction simulated with the computed regression coefficients depicted the restraint on the force generation after stroke compared to the control group. More specifically, the motor modules of the stroke group did not represent a certain sub-force space, a combination of lateral, forward, and upward directions. Our prior study identified this observation to show that more than 40% of recruited stroke participants could not match these targets without compensatory lateral arm rotation (Roh et al., 2013). Moreover, our result extends the findings from Lum et al. (1999) and Reinkensmeyer et al. (2000), which characterized the forward-medial force coupling in a 2D horizontal plane. Aligning with the other colleagues’ work showed that dysfunction of a single muscle (Kutch and Valero-Cuevas, 2011) or a change in a motor module (Inouye and Valero-Cuevas, 2016) could alter the force representation, our findings indicate similar results in the stroke survivors by demonstrating the alteration of motor modules and the resultant limited feasible force direction after stroke. Overall, the findings suggest that the abnormality in the composition of motor modules and their activation appears to have contributed to the performance degradation post-stroke throughout the voluntary isometric reaching task.

This study has a few methodological limitations regarding sample size, EMG recording, motor module identification, and feasible force direction simulation. In this study, there was no sample size estimation performed for both control and stroke participants. However, the number of participants was enough to show the statistical significance of the results. In addition, the electrophysiological activities of only eight major muscles in one side of the upper limb were recorded for motor module analysis. Thus, the presented results do not include information on how other muscles in the upper body, such as the back, trunk, and hand muscles, would contribute to the different phases of the isometric reaching. Particularly, including distal muscles may influence the results, since post-stroke motor modules involving hand and wrist muscles could not be well represented as a simple merging of motor modules recorded in healthy participants during an isometric task (Lee et al., 2013) unlike the case when hand muscles were not included in the UE post-stroke (Cheung et al., 2012). Also, motor module identification from a small subset of muscles via the conventional method using VAF could lead to misestimation of the number of motor modules (Steele et al., 2013). To overcome the risk, the sub-thresholds, diffVAF and mVAF, in addition to gVAF were applied for module number estimation in this study. The recorded EMG from the eight selected muscles could be affected by the cross-talk between EMG channels and other experimental noises, including internal noise of the EMG recording system and motion artifacts, which potentially influence the structure of the motor module. To minimize the cross-talk effect on the EMG signals, we pre-checked the raw EMG signal in real-time after placing the electrodes on UE muscles and confirmed that no muscles generally showed an unreasonable pattern of activation. In order to limit any bias in task performance resulting from motor learning or muscular fatigue, the order of targets was randomized for each participant. For the artifacts, the multiple steps of signal post-processing were adequately adapted. Moreover, unit-variance normalization was applied to the processed EMG to minimize the physiological and anatomical intersubject variability. In the feasible force direction simulation, the quadric terms of the activation coefficient of the motor module (e.g., E Flex and E Ext combined term) were excluded from the multiple linear regression to focus on the contribution of each module on the endpoint force generation.

In addition, this study only includes stroke survivors with severe impairment, so the inference of the findings to the wider post-stroke population needs to be further investigated. Our previous results (Roh et al., 2015) showed that alterations in the shoulder motor module underlying isometric force generation appear prominently in mild and moderate stroke, as in most cases of severe stroke, in an impairment level-dependent manner. Since the findings of this study suggest that alterations in the shoulder motor module contribute to the abnormal end-point force control under isometric conditions, we expect that similar abnormal between-force coupling will also appear in mild and moderate stoke and more frequently as the severity of motor impairment increases.

Potentially, the variability in the FMA score across the stroke participants may have influenced the results of this study. However, Roh et al. (2015) showed the incidences of impaired shoulder motor module were observed in most cases of severe stroke and were positively correlated with impairment level (FMA score). Therefore, we anticipate that the influence of inter-subject variability, in terms of the FMA score, on the results may not be significant, since only the severely impaired stroke participants (FMA score < 26/66) were included in this study. Moreover, Roh et al. (2015) also showed the incidences of impaired shoulder motor module were not correlated with the time after stroke onset, however, an earlier study (Cheung et al., 2012) showed that post-stroke alterations in the motor module were correlated with time after stroke-onset. Therefore, it merits further study to investigate the direct relationship between the time after stroke-onset and our results on the altered force representation.

The findings of this study provide implications for the current neuromotor rehabilitation field. The knowledge of how the muscle activation patterns and their end-point force representations develop during exploratory isometric reaches after stroke may benefit in designing a motor module-based assistive device or robot for rehabilitation (Alibeji et al., 2018; Wei et al., 2020; Wang et al., 2021). In particular, for the patient experiencing a severely restricted movement of the affected limb, the assistive device or robot may be able to predict the patient’s intention more promptly and provide an accurate guide to a target direction by utilizing the modules’ representation of the endpoint force. In addition, the findings from the present study support that modifying the stroke-induced motor modules can be targeted directly to improve motor function and the feasible force direction. For this approach, the modifiability of the motor module needs to be confirmed prior to designing an actual rehabilitation protocol. Lastly, the current study can be extended to investigate the generalizability of the motor module across isometric and movement tasks (Cheung et al., 2009). Since it is yet unclear whether the motor modules observed during the isometric task share a similar composition and onset point with the modules underlying reaching movement, verification of such transferability will justify that a more accessible isometric reaching protocol can substitute for a dynamic reaching protocol in training patients with severe UE motor impairment.

## Data availability statement

The original contributions presented in this study are included in the article, further inquiries can be directed to the corresponding author.

## Ethics statement

The studies involving human participants were reviewed and approved by Northwestern University Institutional Review Board. The patients/participants provided their written informed consent to participate in this study.

## Author contributions

GS completed the data analysis, interpretation, and manuscript writing. SL and RB contributed to designing the study, interpreting the results, and writing the manuscript. AA contributed to manuscript writing and statistical analysis. Y-NW, PR, and WR contributed to data interpretation and writing the manuscript. JR designed the study, collected the data, assisted in data analysis and interpretation, and wrote the manuscript. All authors read and approved the final manuscript.
